# Community-Based Doulas and Medicaid Expansion: A Resource-Based Approach to Support the Well-being of Low-Income Postpartum Women

**DOI:** 10.21203/rs.3.rs-6234727/v1

**Published:** 2025-04-03

**Authors:** Charlotte V. Farewell, Jennifer Gahrns, Julia Pangalangan, Emily Curl, Anna Pangalangan

## Abstract

Background Doula care across the perinatal period may significantly reduce the odds of perinatal mood and anxiety disorders particularly in communities experiencing deprivation. In 2023, SB23-288 was passed in Colorado titled “Improving Perinatal Health Outcomes” which requires doula services to be covered under Medicaid. The purpose of this phenomenological qualitative study was twofold: 1) To examine perceptions of SB23-288 among a purposive sample of community-based doulas who provide care for women experiencing low socioeconomic status (SES), and 2) To explore perceptions among both community-based doulas and low-SES postpartum women themselves, of multi-level resources that are most protective and may mitigate the risk of postpartum mood disorders. Methods A purposive sample of 16 low-SES postpartum women and 9 community-based doulas were recruited to participate in 30–60-minute interviews. Coding followed a constant comparison method and was an iterative process including a deductive, theory-driven approach based on the Conservation of Resources Theory and Socioecological Models and an inductive, data-driven approach. Results Four major themes emerged related to doulas perceptions of strengths and concerns of SB23-288: 1) Improved access to care for low-income individuals, 2) Cautious optimism for Medicaid coverage, 3) Reimbursement-related challenges, and 4) Concern with doula autonomy. Analyses also revealed concordance between doulas and postpartum women’s perceptions of individual-level (e.g., lactation support) interpersonal-level (e.g., family support), and community-level (e.g., navigating healthcare and financial support) resources that promote postpartum mental health. Low-SES postpartum women also shared that knowledge related to postpartum physiology and physical recovery, connections to educational classes to provide peer support, and outdoor exposure were additional resources that promoted their mental health. Conclusions These findings underscore the vital role that community-based doulas play in supporting low-SES postpartum individuals and highlights both the opportunities and challenges presented by the expansion of doula care for under resourced individuals. The alignment between community-based doulas and low-SES postpartum women’s perceptions of multi-level supports emphasizes the importance of holistic, community-centered care and additional training on multi-level and comprehensive resources to meet the needs of low-income clients.

## INTRODUCTION

Doulas are certified professionals who serve as advocates and provide support for pregnant, birthing and postpartum women and their families.(1) The specific responsibilities of a doula vary depending on the patient's needs. Labor and birth doulas specialize in maternal health and assist the care team with following the patient’s birth plan including support navigating physical sensations, birthing positions, breathing techniques, and comfort measures. Doulas may also work with the patient before birth on their birth plan and provide education, as well as advocate for the laboring patient, encouraging accountability with the team of healthcare professionals.(2) Postpartum doulas may provide physical, emotional and informational support to families after birth, including helping to navigate newborn care, breastfeeding, mental health supports, and household tasks.(3)

Doulas can play a significant role in mitigating labor and birth complications.(4) According to the Centers for Disease Control and Prevention (CDC), around 80% of maternal deaths in the U.S. are preventable. Social determinants of health including race, gender, economic status, and cultural background contribute to maternal morbidity and mortality outcomes.(5) For example, black women in the U.S. are more likely to experience adverse maternal health outcomes, such as pre-term birth, death in childbirth, and infant death compared to White women.(6) Within the healthcare team, there are a multitude of gaps, such as patient-provider communication, that contribute to these disparities and prevent comprehensive equitable care. Doulas can help to buffer these gaps, thus buffering maternal and child health disparities.(7)

Importantly, doula care mitigate adverse maternal mental health outcomes, significantly reducing the odds of perinatal mood and anxiety disorders (PMADs). For example, in a propensity score matching study in a large sample of low-income Medicaid recipients across 3 states, women who had received doula care had 57.5% lower odds of PMADs compared to those who had not received doula care.(8) Further, doula care was significantly associated with reduced odds of PMADs even among women with pregnancy complications.(8) In a scoping review on the role of doulas in addressing perinatal mental health and substance use, Haiman et al.(9) found doulas positively impacted mental health, reducing symptoms of anxiety and depression and playing a key role in interdisciplinary teams caring for perinatal individuals experiencing substance use challenges. Similarly, Falconi et al. (8) found that women who received support from a doula solely during labor and delivery showed a 64.7% reduction in odds of PMADs, suggesting that the impact of doula care during the labor, delivery, and postpartum experience may be particularly impactful on postpartum mental health outcomes.

Despite the demonstrated value of doulas in supporting postpartum mental health outcomes, the efficacy of doulas among marginalized communities is less clear. In a randomized controlled trial providing a home-visiting based doula intervention to a sample of primarily African American, Latina, or multiracial women across four communities, no difference in postpartum depression symptoms was observed between the intervention group and the case-management control group.(10) Additionally, Schytt et al. (11) conducted a randomized controlled trial with a bilingual community-based doula care intervention in a sample of migrant women in Sweden and found no significant differences on postpartum depression measures or ratings of birth experience between intervention and control groups. However, in a review of community-based interventions addressing perinatal mental health, over half of the interventions found to be effective or partially effective focused specifically on low-income populations with racially and ethnically diverse participants, suggesting that community-based interventions, such as doula care, may be particularly important in populations experiencing maternal health disparities. (12)

In 2023, the Colorado General Assembly passed SB23-288 titled “Coverage for Doula Services”, establishing reimbursement for doula services provided to recipients of Health First Colorado (HFC), the state’s Medicaid program, beginning no later than July 1st, 2024. Furthermore, the bill initiated a stakeholder engagement process aimed to: 1) Promote expansion and utilization of doula services for pregnant and postpartum Medicaid recipients who face disproportionately greater risk of poor birth outcomes, 2) Solicit feedback regarding the establishment of approved doula certification pathways and billing processes for doula services, and 3) Create a doula scholarship program to grant funds to individuals without sufficient financial resources to complete doula training and certification programs.

The coverage of doula services under Medicaid is especially monumental as Medicaid is the single largest payer of maternity care in the U.S.(13) Other states have struggled with participation in their respective Medicaid expansion programs for doula care due to provider shortages and inadequate coverage and reimbursement structures, which range from 4 to 12 covered visits and $770 to $1500 reimbursement limits. (14) Florida, Oregon, and Minnesota have had active programs for a combined 10 years, but collectively have only serviced a total of about 1,000 births.(14) Doula certification is not regulated; however, several national training and certification bodies exist along with many independent and local training agencies that will award a title of Certified Doula upon completion of their programs. (15, 16) Lack of an official certification process has been a consistent challenge for states introducing policies authorizing Medicaid reimbursement for doula services, leaving them to determine relevant qualification standards for participating doulas.(17, 18) Though there is significant variation in doula certification programs, core competencies generally focus on perinatal support; childbirth education; non-medical comfort measures; labor support techniques; postpartum/recovery support; feeding/lactation support; newborn/infant care; anatomy of pregnancy, childbirth, and postpartum; family/partner support; developing a community resource list; trauma informed care; and diversity, equity, and inclusion/cultural sensitivity.(15)

Importantly, with the passing of SB23-288 in Colorado, ensuring that doula care is aligned with the unique needs and resources of socioeconomically disadvantaged communities is critical to help to buffer exposure to adverse social determinants of health (SDoH) and mitigate postpartum mental health disparities. For example, a recent systematic review found that social support, food insecurity, and housing were the most pressing needs mentioned among low-income perinatal individuals; these SDoH uniquely contribute to PMADs.(19) Doulas are well situated to provide support as well as connect marginalized individuals to community resources that will help to foster a positive perinatal experience. However, exploration of the lived experience of perinatal women experiencing low socioeconomic status (SES), and how their identified needs and supports align with community-based doula care, is lacking. The purpose of this phenomenological qualitative study was twofold: 1) To examine community-based doulas insights on SB23-288 in Colorado, and 2) To explore perceptions among both community-based doulas that provide care for low-SES women and low-SES postpartum women themselves, of multi-level resources that are most protective and may mitigate the risk of postpartum mood disorders. These findings provide recommendations on how to better support doula's in their work with low-SES perinatal individuals and ultimately help to mitigate maternal morbidity and mortality disparities experienced by underserved families.

### METHODS

## Research Team Positionality and Reflexivity

Our research team brings a rich background in perinatal mental health and an in-depth understanding of risk and protective factors associated with the perinatal experience. Author 1, who led this study and conducted the majority (75%) of all postpartum interviews, has extensive qualitative expertise reflected by numerous mixed-methods and qualitative publications in peer reviewed journal articles related to perinatal mental health. Author 1's positionality as a researcher and personal experience with doula support provided a unique perspective, balancing empathy with critical inquiry. The remaining authors represent master's and doctoral-level students who are committed to studying population mental health and well-being, with a particular focus on promoting the intergenerational transmission of resilience. Authors 2, 3, and 4 conducted the remaining postpartum and doula interviews and led analyses, which were double coded and reviewed by Author 1 following the methodology described below. A relationship with the participants was not established prior to conducting this study.

### Participants

All procedures were approved by the Colorado Multiple Institutional Review Board (IRB #: 22-1725). The sample for this qualitative study came from a larger mixed-methods parent study that recruited pregnant women in Colorado from two healthcare systems using non-probability purposive sampling techniques between May 2023 and December 2023 (Mothers Optimizing Resources Everyday (MORE); n = 223). Eligibility criteria included > 6 weeks pregnant, at least 18 years of age, English- or Spanish-speaking, receiving prenatal care at one of six health care clinics in the Denver metro area, and experiencing socioeconomic disadvantage. Participants were classified as experiencing socioeconomic disadvantage if they endorsed low income (defined in five ways: Medicaid-recipient, participation in federal assistance programs, household income < 250% FPL, no- or partial-insurance, subjective experience of high financial stress), if they endorsed low educational attainment (less than college degree), or if they endorsed both factors.

### Procedures

The study consent and surveys for the parent study (MORE) were administered via Research Electronic Data Capture (REDCap).(20) REDCap is a secure, web-based application designed to support data capture for research studies. As part of MORE, pregnant women were asked to complete up to three additional surveys: 3rd trimester, 6-weeks postpartum, 12-weeks postpartum. Quantitative data is not shared in this paper. The surveys included a yes/no question about willingness to participate in an interview during pregnancy and during the 6-weeks postpartum data collection timepoint. For the current qualitative study, the study team reached out to those who agreed at 6-weeks postpartum, by phone or email according to their preference, to schedule an interview.

Community-based doulas who work with low-SES women were recruited using purposive sampling from community partners and through word of mouth. Though doulas are often paid for privately by the patient or the patient's family, community-based doulas are typically supported and paid by an organization or program that is specific to the patient's community.(13) Community-based doulas can provide extended services or connections due to their role in an established organization. (13) Their services and jobs can also be more personalized for the individuals' culture, and they are often able to spend more time with the patients. To recruit community-based doulas for the study, the recruitment flyer was shared with leadership from community partners and leaders disseminated to the organizations' doula networks. If individuals were interested in participating in an interview, they contacted the study team directly and an interview was scheduled. Interviews were conducted until the study team deemed that saturation was reached. Both postpartum women and doula interviews were held over the phone or videoconference using Zoom software and ranged from 30 to 60 minutes. Individuals received a $20 Amazon gift card for participating.

### Instruments

Following a phenomenological approach, the interview guides were focused on investigating the lived experiences of individuals to understand how they perceive and interpret their experiences related to perinatal supports. The postpartum interview guide was framed around the conservation of resources (COR) model and socioecological model (SEM). The COR theory outlines how personal and environmental capacities support well-being in the face of adverse social determinants.(21) Multi-level resources can be particularly protective for individuals experiencing adverse social determinants such as socioeconomic disadvantage. Likewise, the SEM addresses the central role of individual- (e.g., knowledge, self-efficacy), interpersonal- (e.g., social support), community- (e.g., social capital) level resources that can promote mental health and well-being.(22) Among individuals facing socioeconomic disadvantage, supplementing multi-level resources during pregnancy may result in a more positive prenatal experience and better birth and postpartum outcomes. For example, mindfulness has been leveraged to help protect the mental health of populations facing socioeconomic disadvantage.(23) Likewise, social resources such as different types and sources of social support can protect individuals from stressors and foster well-being during pregnancy. (24–26). Community-level resources, such as social capital, can buffer the effect of adversity on maternal mental distress. (27) The interview guide included questions regarding these multi-level supports as well as how accessing these resources helped women cope with external stressors and supported their well-being. For example, postpartum women were asked the following questions: "*In general, what activities or behaviors have been helping you to feel good?" (individual resources), "Who has helped support you thus far in the postpartum period and how have they helped?" (social resources), and "What has your relationship been like with your doctors (both OB and pediatrician) and how has that impacted your well-being?" (community resources)*.

The doula interview guide included three sections: 1) Perceptions of Medicaid expansion for doula coverage in Colorado, including anticipated barriers, 2) Experiences working with low-income clients and topics that they cover most frequently with these clients, and 3) Primary challenges low-income clients are facing with respect to having a positive birth experience. For example, doulas were asked, "*What has your experience been like providing care for low-resourced (low income) individuals? What are the barriers and facilitators to working with this population and supporting them to have a positive birth experience?*" and "*Thinking back to when you got trained to be a doula, what topics or content areas do you discuss most frequently with your low-income clients?"*

## Data Analysis

Sixteen (16) semi-structured interviews with postpartum women and 9 semi-structured interviews with community-based doulas were conducted and included in the final analysis. Interviews were audio recorded with participants' consent and transcribed verbatim utilizing Microsoft Word's transcribe feature followed by manual verification and correction referencing the original recordings. All interview transcripts were imported into ATLAS.ti version 23.2.1. Coding followed a constant comparison method and was an iterative process including a deductive, theory-driven approach and an inductive, data-driven approach. The structural coding scheme was a priori, categorized into individual-, interpersonal-, and community/provider-level resources that may support positive coping behaviors and postpartum experiences. *A priori* codes allowed the study team to consistently align with the COR theory and SEM and begin theory building with themes within these domains. There were two key reviewers and a third reviewer who double-coded 4 of the 16 interviews. All three coders and the principal investigator met weekly to discuss questions and reach consensus on any disagreements that arose. Once coding was complete, the team reviewed codes and identified key themes. To ensure the trustworthiness of the data collection, analysis and interpretation, the team triangulated the data across interviews during weekly meetings and used reflexive memoing. All data collection and analysis procedures were documented, including an iterative codebook based on coding and recoding of the data and peer examination of data analyses.

## RESULTS

**[Insert Table 1 Here]**

Table 1 displays demographic characteristics of the sample of postpartum women and community-based doulas that participated in this study. Among the sample of postpartum women (n=16), the average age was 29 (SD=6.1) and the average number of weeks postpartum was 4.4 (SD=2.2). Approximately half of the sample was Hispanic (53.3%), 56.3% were White and 31.3% were Black, 62.5% reported less than a college degree, 50% were married, 50% experienced a high-risk pregnancy, 25% were first time parents, and 42.9% were currently receiving assistance from the Women, Infants, and Children (WIC) program. Among the sample of nine community-based doulas, just under half (45.5%) were Hispanic, 77.8% were White, and all held a college degree.

**[Insert Table 2 Here]**

When asked about perceptions and implications of the new Medicaid policy, four major themes emerged and are described with representative quotes in Table 2: 1) Improved access to care for low-income individuals, 2) Cautious optimism for Medicaid coverage, 3) Reimbursement-related challenges, and 4) Concern with doula autonomy.

#### Strengths

Generally, doulas in this sample felt cautiously optimistic about SB23-288. Doulas hoped that Colorado's Medicaid reimbursement policy would allow them to serve low-income individuals in a way that is financially feasible. Prior to this Medicaid benefit, doulas often dealt with strain around their desire to support low-SES individuals while ensuring they can financially support their own families. The implementation of this Medicaid policy can support doulas in extending their care into communities that they could not reach previously, which may have downstream impacts on material-child health outcomes.

#### Concerns

While doulas were excited about the improved access to care, they also identified several anticipated barriers it may present. Doulas expressed concerns around the increased regulatory authority that the state will have through this program. State regulation of reimbursement rates and other standards such as training requirements could present challenges for doulas. For instance, the reimbursement rates may not be sufficient compared to what a doula was charging independently. Additionally, given the current variety in doula trainings and certifications in Colorado, the requirements for doulas to be approved providers under this new Medicaid law may not include all practicing doulas. Doulas also mentioned that there could be language or cultural barriers that prevent some practicing doulas from completing the applications and trainings to become eligible for Medicaid coverage. Historically, the doula profession has served in part to advocate for clients within systems of oppression. The restrictions enacted in the Medicaid law may interfere with doula's autonomy and the ways in which they advocate for clients.

Overall, doulas recognized the potential benefit of the Medicaid policy but held concerns about the practical applications.

**[Insert Table 3 Here]**

Community-based doulas were asked about their experiences working with low-SES clients and topics that they cover most frequently. Low-SES postpartum women were asked about the supports they have and want to better support a positive postpartum experience. Multi-level resources identified by both groups were categorized by individual-, interpersonal-, and community-level supports following the SEM (See Table 3).

#### Individual-level resources

At the individual-level, both doulas and postpartum women expressed the need for lactation support. One doula shared, *"A lot of people want to be successful at breastfeeding. And the resources that they get are so conflicting from a pediatrician, from the lactation consultant, to what their grandma told them to do."* Doulas also identified mind-body practices and sleep support as crucial needs for low-SES perinatal individuals. Doulas highlighted the practical benefits of mind-body practices for preparing the body and mind during the perinatal period, though this resource was not mentioned among the postpartum individuals in this sample. Postpartum women did discuss lack of sleep, but they were less explicit about having or needing access to more resources for sleep support. More commonly, postpartum women emphasized wanting more education and resources related to the postpartum recovery process, including a focus on physiologically what is happening in the body. The slight differences in themes identified by doulas and postpartum women in this sample may indicate a need to prioritize education regarding postpartum recovery, particularly when working with low-SES perinatal populations.

#### Interpersonal-level resources

At the inter-personal level, social support was identified by doulas and postpartum women as a crucial resource. Doulas facilitate social support for their clients through connecting clients to resources and navigating their existing social relationships and networks. One doula described it this way:

*[…] before baby is even born, we focus on creating a postpartum abundance plan and that looks like sitting down and talking about when baby is here, what is the support that we need and how can we make boundaries to communicate with say, like our parents or our friends in what we need. And so, we scaffold that before it gets stressful and complicated.*

Doulas and postpartum women acknowledged the value of emotional support from family and friends. Though both groups discussed the importance of familial support, the postpartum women discussed how access to a peer group, particularly connections with women at similar life stages, was most protective with respect to their postpartum mental health. The themes of familial and friend support from both groups were the most frequent and salient in these data.

#### Community-level resources

At the community-level, both doulas and postpartum women identified the value of needing support with navigating healthcare systems and providers. Doulas highlighted the importance of advocacy and effectively fostering positive interactions with care teams. Postpartum women wanted more continuity of care in their perinatal care experience. This is a critical role of doulas and an area of capacity building needed within low-income populations. Given that this was a low-income sample, financial support resources emerged as an essential community-level resource. Community doulas acknowledge and help to address the financial complexities that low-income postpartum women face:

*[…] especially with people who may be returning to work sooner, not able to navigate kind of the family leave act, not having a lot of supports or resources in place, being concerned about not having you know, six months of income saved up to pay bills and things like that, spending more time navigating those things can help reduce anxieties and have them feel like they know where they can turn.*

While the postpartum women in this sample shared a level of financial strain, the theme was more salient for doulas. Regarding community-level resources, postpartum women expressed a need for educational classes as well as the benefits of outdoor exposure to support their well-being.

#### Summary of Qualitative Findings

Analyses revealed concordance between doulas and postpartum women in unique individual-level (e.g., lactation support), interpersonal-level (e.g., social supports), and community-level (e.g., navigating healthcare) resources. Low-SES postpartum women also emphasized how additional knowledge related to postpartum physiology and recovery (both mental and physical), connections to educational classes to foster peer support, and benefits of outdoor exposure were especially promotive of their mental health suggesting the need to further emphasize this information in doula certification programs thus increasing these supports.

### DISCUSSION

The purpose of this phenomenological qualitative study was to explore perceptions of expanded Medicaid reimbursement for doula services among a purposive sample of community-based doulas and to examine the alignment between community-based doulas and low-SES postpartum women's perceptions of multi-level resources needed to promote a positive prenatal, birth, and postpartum experience. With respect to the first aim, this sample of community-based doula's shared strengths as well as concerns related to SB23-288. Doulas also shared numerous multi-level resources that support the mental health and well-being of their low-income patients such as knowledge around breastfeeding, sleep practices, and mind-body supports (individual-level), how to access and bolster informal social support systems, particularly related to family supports (interpersonal-level), and navigating interactions with healthcare providers and accessing financial resources (community-level). Low-SES postpartum women highlighted many of these same supports as crucial to their postpartum mental health and well-being with some additional key resources noted.

The central concerns shared by doulas in this sample regarding the implementation of the new Colorado Medicaid law related to regulations and standards, diminished autonomy, and unreliable payment structures. This sample discussed the historically independent nature of the doula profession and the importance of professional autonomy for doulas, particularly in an oppressive healthcare system. There is an understanding that with policies like SB23-288, law makers and Medicaid professionals will be responsible for setting the standards for certified doulas which may present barriers for community-based doulas specifically.(28) As non-medical providers, billing to Medicaid imposes a high bureaucratic load on community-based doulas who work independently or as part of a collective of doulas.(13) Medicaid reimbursement also introduces a delay in payment as claims are processed, presenting another barrier for low-income community-based doulas. Additionally, services extending beyond doula visits, such as connection to community resources, are not covered by the reimbursement structure.(13) Finally, the community-based doulas in this sample aligned with concerns expressed among Colorado stakeholders regarding Medicaid's mandatory reporting requirements; of the community members surveyed through the bill's stakeholder engagement process, 19% reported that a Mandatory Reporter requirement would stop them from utilizing a doula and an additional 32% were unsure how this requirement would impact their choice.(14) Though the doula community agrees that SB23-288 can increase access to life-saving care and help to diminish the perinatal health disparities present in U.S. maternal healthcare, these findings suggest additional work is needed to address these concerns and better support the doula community.(29)

Doulas shared that breastfeeding, mind-body practices and sleep-related strategies (individual-level), connection to social support systems, particularly family support, (interpersonal-level) and advocacy with care providers and connection to financial supports (community-level) were the most common supports that they provided for their low-income perinatal clients. A recent qualitative study sought to assess doulas' experiences delivering lactation education to racially/ethnically diverse, the majority of whom were on Medicaid. (30) Findings suggested that doulas emphasized the need for more lactation education, specifically to support their work with low-income families. Connecting women to social supports was a commonly shared topic among doulas in this sample. A systematic review highlighted the buffering effect of social support on mitigating adverse SDoH and resulting perinatal health disparities (19); doulas are uniquely positioned to foster connection and belonging across the perinatal period. Finally, connection to financial resources and advocacy with care providers were frequently mentioned themes. Doulas often provide resources outside of typical birth roles, including connections to the Special Supplemental Nutrition Program for Women, Infants, and Children (WIC) benefits and Healthy Start programs. (31)They also support their low-SES clients' ability to navigate and advocate for themselves while interacting with care providers.(31)

Among this sample of low-SES postpartum women, information related to breastfeeding and postpartum physiology and recovery; emotional support from family members and friends; and provider navigation, access to educational classes and outdoor exposure were the most frequently shared resources that enhanced their mental health. These individual-level resources are echoed in a recent qualitative study of 54 low-income postpartum women which found that postpartum care and education related to breastfeeding, infant care, and mental and physical health recovery were cited as some of the most important topics to support maternal well-being.(32) At the interpersonal-level, numerous studies have identified the importance of diversified types and sources of social support across the perinatal period to effectively mitigate stressors that arise from the challenging transition to parenthood.(33) At the community-level, women in this sample cited the need for support related to healthcare provider access and continuity of care; low-SES postpartum women may face challenges related to scheduling appointments, insurance limitations, lack of provider continuity, and knowledge gaps.(34) A unique protective resource cited by this sample of postpartum women was exposure to the outdoors. Though an extensive body of literature cites the benefits of outdoor exposure with respect to mental and physical health disparities (35, 36), only three studies have investigated nature-based therapeutic strategies among postpartum women, highlighting an opportunity for further inquiry and doula support. (37)

**[Insert Table 4 Here]**

The findings of this study, in conjunction with existing work, have several implications for program development, research, and policy (See Table 4). Based on observations from other states that have implemented Medicaid coverage for doula care, it is recommended to have doulas, specifically community-based and doulas of color, as part of the decision-making process for Medicaid programs. They should serve as expert advisors as the program continues to be evaluated and improved. (38) Doulas can also assist in revising the registration process to ease the burden of obtaining Medicaid reimbursement. They can advise on regulations, systems of payment, training requirements, and focus areas for research. (2) Another recommendation based on these findings is to adjust the certification requirements for involvement in Medicaid benefits.(28) For example, many community-based doulas receive their training from a community organization as opposed to a national organization. Medicaid may not recognize these community certification programs which results in these doulas being ineligible to receive payment through Medicaid. (28) Findings from this study align with existing evidence that emphasizes the need for increased education and awareness related to both the benefits of doula care as well as accessing doula support for low-income perinatal women and healthcare professionals alike. (2) The perspective of doulas as well as low-income perinatal women, is key in implementing services that will accomplish the goal of limiting maternal health disparities and increasing access to life-saving care.

Though this study has numerous strengths it is not without limitations. First, the participants represents a small, purposive sample which may limit the generalizability of these findings and increase the potential for overlooking alternative diverse perspectives. Second, there is variation among the postpartum women in this sample with respect to exposure to adverse social determinants of health (e.g., ~½ are receiving WIC services) and socioeconomic disadvantage. It is important to acknowledge the gradient of exposure to adverse SDoH and though all individuals met the inclusion criteria based on low-SES, it is likely that they are experiencing variation in external stressors that could impact their responses to multi-level supports that may be most protective. Finally, the research teams' subjective experiences and biases, as is the case in all qualitative research, may impact the interpretations and conclusions drawn from these data.

## Conclusions

In summary, findings from this qualitative study underscore the vital role that community-based doulas play in supporting low-SES postpartum individuals and highlights both the opportunities and challenges presented by the expansion of doula care for under resourced individuals. While Medicaid coverage of doula care has the potential to improve access to these supports and help mitigate perinatal-related health disparities, concerns regarding reimbursement and autonomy must be addressed to ensure its success. The alignment between community-based doulas and low-SES postpartum women's perceptions of multi-level supports emphasizes the importance of holistic, community-centered care, with an additional focus on promoting postpartum physiological recovery, connection to educational classes, and outdoor exposure as key protective factors with respect to PMADs and postpartum well-being. Moving forward, policies and healthcare systems should prioritize reimbursement and administrative support for community-based doulas to access Medicaid reimbursement and ensure doula training certification programs encompass a broad focus on multi-level and comprehensive resources to meet the needs of their low-income clients.

## Abbreviations

**PMADs = Perinatal Mood and Anxiety Disorders**

**SDoH = Social Determinants of Health**

**SES = Socioeconomic Status**

**MORE = Mothers Optimizing Resources Everyday**

**SEM = Socioecological Model**

**COR = Conservation of Resources**

## Declarations

**Ethics approval and consent to participate:** All procedures were approved by the Colorado Multiple Institutional Review Board (#22-1725) in accordance with the Declaration of Helsinki. All individuals consented to participate in this study.

**Consent for publication:** n/a

**Availability of data and materials:** Data is available from the authors upon request.

**Competing Interests:** The authors declare that they have no conflict of interest.

**Funding:** This work was supported by the National Institute on Minority Health and Health Disparities (5K01MD016928-02) and (NIH/NCATS Colorado CTSA Grant Number UL1 TR002535)

**Authors Contributions:**CVF lead the conceptualization of this project, most of the writing, conducted 75% of the data collection activities and oversaw the described data analyses. JG, JP, and EC supported the data collection and analyses, as well as drafting and editing the paper. AP reviewed the data and supported drafting and editing processes. All co-authors reviewed the final manuscript.

**Acknowledgements:** We acknowledge and thank all doulas and postpartum women who participated in this study.

**Clinical trial number:** not applicable

**Running Head**: Doula care, Medicaid Expansion, and Multi-level Resources

**Word count: 4830**

## Tables

Table 1. Sample Demographics (n=16 Postpartum Women; n=9 Community-Based Doulas)

| | Postpartum Women (n=16) | | Doulas (n=9) | |
|---|---|---|---|---|
| | m | sd | m | sd |
| Age | 29.0 | 6.10 | 30.5 | 6.7 |
| Number of Weeks Since Giving Birth | 4.4 | 2.20 | n/a | |
| Years of Doula Practice | n/a | | 7.5 | 5.2 |
| | n | % | n | % |
| Ethnicity | | | | |
| Non-Hispanic | 7 | 46.7 | 5 | 55.5 |
| Hispanic | 8 | 53.3 | 4 | 45.5 |
| Race (select all) | | | | |
| White | 9 | 56.3 | 7 | 77.8 |
| Black | 5 | 31.3 | 2 | 22.2 |
| American Indian or Alaskan Native | 1 | 6.3 | 0 | 0 |
| Asian or Pacific Islander | 2 | 12.3 | 0 | 0 |
| Other Race | 1 | 6.3 | 0 | 0 |
| Education | | | | |
| Some High School | 4 | 25.0 | 0 | 0 |
| Completed High School | 2 | 12.5 | 0 | 0 |
| Some College | 4 | 25.0 | 0 | 0 |
| Completed College | 5 | 31.3 | 5 | 55.5 |
| Beyond College | 1 | 6.3 | 4 | 45.5 |
| Marital Status | | | | |
| Married | 8 | 50.0 | n/a | |
| Single and never married | 1 | 6.3 | n/a | |
| A member of an unmarried couple (partnered) | 4 | 25.0 | n/a | |
| Separated | 2 | 12.6 | n/a | |
| Other | 1 | 6.3 | n/a | |
| Experienced a High Risk Pregnancy | | | | |

| | | | |
|---|---|---|---|
| Yes | 7 | 50.0 | n/a |
| No | 6 | 42.9 | n/a |
| Don't Know | 1 | 7.1 | n/a |
| First Time Parent | | | |
| Yes | 4 | 25.0 | n/a |
| No | 12 | 75.0 | n/a |
| Currently Receiving Support from WIC program | | | |
| Yes | 6 | 42.9 | n/a |
| No | 8 | 57.1 | n/a |

**Table 3. Protective Multi-level Resources to Mitigate PMADs for Low-SES Perinatal Women Reported among Postpartum Women (PPW) and Community-based Doulas**

| Table 2. Community-Based Doula's Perception of Strengths and Concerns related to Expanded Medicaid Coverage of Doula Services | | |
|---|---|---|
| **Key Themes** | **Description** | **Representative Quotes** |
| Improved access to care for low-income individuals | Participants shared that supporting doulas financially through Medicaid will help to improve access to doula care for low-income perinatal individuals. | "Whether I'm doing it pro bono or not, this is a way that I can get paid […] we have all wanted to work with different socioeconomic people more and trying to figure that out, the Medicaid benefit has made a way for that" |
| | | "I would love for everyone to have access to a doula that wants one, but I also have to be able to contribute to my family financially, and so it does limit the amount of clients that I can take that are free or reduced rate" |
| Cautious optimism for Medicaid coverage | Participants shared a general sense of hope and optimism regarding the bill, yet all indicated some type of hesitancy in their statements. | "I think there's two sides to it. I'm excited that Medicaid will cover doula care because I feel like it opens up an avenue for people to have support" |
| | | "I think that's really wonderful, and I think we rolled it out as a state too quickly and we haven't thought through some things. And I think that that is going to impact the ability for clients to feel like they have true access to that" |
| Reimbursement-related challenges | Participants expressed concern around the regulatory authority of the state in determining reimbursement rates and setting training requirements. | "If an insurance company decides that we are only worth X amount of dollars and they're unwilling to pay our fee or a more reasonable fee, I think one, that's still going to leave a lot of us who do this, to be unable to serve people on Medicaid. But two, I think it will also tell the community at large that we are not worth what we charge" |
| | | |
| | | "The restrictive capacity of the insuring bodies is going to then restrict who has access to that reimbursement" |
| Concern with doula autonomy | Participants are concerned about state regulations affecting their independence and advocacy. Several participants emphasized how the independent nature of the doula profession is essential to | "We have no medical responsibility, our job is to work for the client, for the birthing family, and when you start slipping into national certification that needs to be covered by Medicaid it just starts taking away the power from the laboring family and putting it more into the hands of these certifying organizations and it's going to |

| | | |
|---|---|---|
| | advocating for their clients within systems of oppression. | make it more complicated for the true birth sovereignty and advocacy to be a part of births, especially for vulnerable populations”<br><br>“There's a really fine line you dance over as a doula when it comes to advocacy and empowerment and that dance has to be done in a way that acknowledges systems of privilege and oppression. I think anytime there is a governmental process that has to be applied to something that has thus far been a little bit more unregulated, there needs to be due diligence done to think about how do we do that in a way that honors what this profession has existed as.” |

| Resource Level and Key Theme | Source<br><br>(Doula, Postpartum Women (PPW)) | Representative Quotes |
|---|---|---|
| **Individual-level Resources** | | |
| Lactation support | Both | "I would say that breastfeeding is the biggest thing they worry about and or need support with because obviously formula is very expensive" (doula) |
| | | "I also scheduled a consultant. So I went to her several times. To work on lactation because I got mastitis and a clogged duct that I had to work through so I my emotions were everywhere on that as well." (PPW) |
| Mind-body practices | Doulas | "Mind-body connection is crucial when it comes to a birth experience that feels personal, that feels connected and not detached to the birthing person" (doula) |
| | | "[…] preparing your body prenatally that you know, if you want low interventions, then you can't just show up that day having not done anything to prepare your body" (doula) |
| | | "I think the yoga and the movement and the breathwork piece is really good for helping pregnant individuals feel into their body as it is growing and sort of have a connection and have a chance to connect with their baby" (doula) |
| Sleep support | Doulas | "I try to really drive home to them the importance of educating themselves on sleep patterns, newborn sleep patterns […] they are still accustomed to a very different rhythm, a rhythm in the womb where it's dark, it's quiet" (doula) |
| | | "[…] it's not as easy as sleep when baby sleeps. I don't ever say that and just really try to remind them about the importance of having some support, some rhythm, routine, schedule […] I think parents go into it with this expectation, sometimes they're going to be able to sleep when baby sleeps or just behave like your adult sleep rhythms" (doula) |
| | | "And talking them through well, here's the norm. Here's where we're at developmentally, and here's what we can encourage. And some of it is just setting routine […] Babies love consistency and when you have those pieces together and you understand and you're encouraging their developmental milestones and you give good structure, then babies sleep" (doula) |
| | | "[…] just the general amount of sleep that you need for mental health and well-being, so the four hours of sleep for four days in a row is really important and something that I talk about and how to make a sleep plan to emphasize how to have the best health, mental health so that you can be your best self and parent. So sometimes that looks like setting up a plan with a partner if there's a partner involved or how to you know, facilitate the best sleep for you and baby" (doula) |

| | | |
|---|---|---|
| Post-partum Physiology and Recovery | PPW | "So just trying to adjust to that um and then I think I had an abdominal muscle tear which kind of made my recovery a little bit harder than any of my previous labors." (PPW) |
| | | "Like the recovery process, I don't know, I've definitely had people like oh like rest your body for like 6 weeks you have to like go do nothing. And then me like being an active person like by like 3 weeks after I wanted to get out and do stuff. And so knowing like what's best for your body." (PPW) |
| | | "I wish I would have read about just the recovery and like the mental state fluctuations." (PPW) |
| **Interpersonal-level Resources** | | |
| Familial support | Both | "If you can convince the matriarch of the family, and I feel like that's in both black and brown communities, whoever the matriarch or the boss is, and really create a relationship with the pregnant person and then say, what's your relationship like with your partner, or with your mother, how would you feel about pulling them into a visit to get to know me, so they understand who I am, so they understand this better, has always worked for me. It's bringing in a moment for people don't feel excluded, so they don't feel like someone is taking over a spot that they have" (doula) |
| | | "[…] familial support is incredibly important no matter what it looks like, and so with every single client we talk about, like who are the people that you consider family and how are they showing up for you both in pregnancy, during birth, and in the postpartum time. And I talk a lot about if there is disconnection between what you want them to show up like and what they're showing up like now, please utilize me [to bridge that gap]" (doula) |
| | | "I encourage them to have a support person outside of a doula and then really doing most of our prenatal sessions together if possible" (doula) |
| | | "I think definitely my husband has been a pretty good support. Mostly just like him and my mom." (PPW) |
| Friend support | PPW | "I think it's really helpful to find like other women not struggling but feeling the same way because it almost eliminates the stigma or like, OK, you're not alone." (PWW) |
| | | "I wish that maybe I had like a friend that went through this." (PPW) |
| **Community-level Resources** | | |
| Navigating healthcare systems and providers | Both | "How to best engage in what might feel like a confrontational conversation with a medical provider […] having like an empowerment and advocacy conversation and reminding people what it looks like to use nonviolent communication" (doula) |
| | | "[…] the biggest piece really is teaching them how to advocate for themselves and to ask questions that this is their experience and |

| | | |
|---|---|---|
| | | that providers do this all day, every day, and so sometimes they don't remember this is a birthday party for you all" (doula) |
| | | "I'm following up with the midwives, but I honestly have felt a little bit like that care is disjointed." (PPW) |
| Financial support resources | Both | "[...] When it comes to my low-income clients, I'm also adding in food stamps, helping them find their local WIC office, because a lot of them don't even know that they can start receiving like in their pregnancy" (doula) |
| | | "I make sure to always tell my clients about their hospital's lactation support groups, postpartum support groups" (doula) |
| | | "Actually if there was more financial help, umm, I know there's a lot of help for families who don't make a lot of money..." (PPW) |
| Educational classes | PPW | "We took a lactation course or had a class with a lactation consultant on breastfeeding and the benefits of it and the different types of latching, which I thought was fantastic" (PPW) |
| | | "Having those classes, giving us the information on medicated, unmedicated, waterbirth would be great for everyone I think" (PPW) |
| Outdoor exposure | PPW | "I think there's something just about like being outside that's like resetting." (PPW) |
| | | "And then like walks every single day, which has been nice and super helpful." (PPW) |

**Table 4. Recommendations on how to Expand Doula Care among Perinatal Women Receiving Medicaid Benefits**

| Theme | Recommendations |
|---|---|
| Mitigate confusion and increasing enrollment in Medicaid-covered doula services | • Increase awareness of doula care among patient population and other healthcare workers and policy makers<br>• Increase education related to Medicaid process for doulas and patients |
| | • Have dedicated Medicaid employees to help with registration |
| Provide a livable wage for doulas | • Pay doulas a salary instead of by birth, pay doulas for their hours outside of the birth as well as on-call hours, offer benefits such as healthcare |
| | • Classify doula care as a USPSTF preventative service and/or using CMS approval for reimbursement |
| | • Increase research on frequency of visits, how doulas spend their time, optimal reimbursement rates |
| | • Have compensation structures based on client-centered practices |
| Prioritize the voice of community-based doulas, doulas of color, and importance of culturally competent care | • Include community-based certification programs in the eligibility requirements, decreasing racial bias<br>• Invite doulas, specifically community-based doulas and doulas of color, to serve as advisors on the policy<br>• Partner with community organizations for equitable care |
| Improve the structure of the law to fit the purpose of the doula profession and the perspectives of doulas | • Increase research and incorporate the voice of doulas<br>• Improve relationships between doulas and Medicaid agencies, hospitals, policy makers<br>• Fund community-based doula organizations |
